# Herbal medicines and kidney; friends or foes?

**Published:** 2014-01-01

**Authors:** Azam Asgari

**Affiliations:** ^1^Medical Plants Research Center, Shahrekord University of Medicine Sciences, Shahrekord, Iran; ^2^Department of Physiology, Faculty of Medical Sciences, Tarbiat Modares University, Tehran, Iran

**Keywords:** Herbal Medicine, Nephropathy, Renal failure

**Implication for health policy/practice/research/medical education:**

As one third to one half of all pharmaceutical drugs was originally derived from plants, the current regulation of herbs does not ensure that available products are safe or not for renal disease.

Herbal products have been widely used around the world since ancient times. Herbal products are complex of organic chemicals that may come from any naive or processed part of a plant, including leaves, stems, roots, flowers, and seeds. Herbs are described as dietary supplements, and manufacturers can therefore produce, sell, and market herbs without first demonstrating safety and efficiency, as is required for pharmaceutical drugs. Many different side effects have been reported owing to active components, pollutant, or interplay with drugs (1-2). The safety of using most herbs with drugs is not well established. Some herbs are recognized to interact with pharmaceutical drugs, while most of this information comes from case reports rather than systematic investigations (3). Many different side effects to herbs have been reported, containing effects from biologically active constituents from herbs, side effects caused by herb–drug interactions or contaminants. Case reports of nephropathy caused by the use of certain Chinese herbs are popular. A specifically morbid case series describes 105 patients in Belgium who had been taking a Chinese herbal product for weight loss and developed nephropathy caused by the herb Aristolochia fangchi (4). While many herbs contain pharmacologically active compounds, certain herbs such as ephedra may cause side effects through excessive biological effects (5). Unfortunately, the true frequency of side effects for most herbs is not known because most have not been tested in large clinical trials. The potential for toxicity from certain herbs is compounded by the frequent use of misleading marketing information.Three groups of herbs can be classified from the safety point of view. 1) Some herbs contain near pharmaceutical concentrations of poisonous constituents such as Arnica spp, Aconitum spp, Atropa belladonna and Digitalis spp. These should not be prescribed by unqualified persons. The second groups of medicinal plants are the ones with powerful actions. These herbs are safe under appropriate conditions. The third is an idiosyncratic grouping of herbs which have been alleged to exhibit specific kinds of toxicities. The best known are the hepatotoxicity of pyrrolizidine-alkaloid-containing plants such as Comfrey, Dryopteris, Viscum, and Corynanthe (6).

Pregnancy is also a condition which should be considered as a time of “contraindication” to taking herbal medicines. The evidence of teratogenicity in humans arising from herbal remedies is rare, but since such evidence would be hard to come by, it is better to be avoided during pregnancy.

## Author’s contribution

AA is the single author of the manuscript.

## Conflict of interests

The author declared no competing interests.

## Ethical considerations

Ethical issues (including plagiarism, data fabrication, double publication) have been completely observed by the author.

## Funding/Support

None.
